# Integrated Physiological and Metabolomic Analyses Reveal the Differences in the Fruit Quality of the Blueberry Cultivated in Three Soilless Substrates

**DOI:** 10.3390/foods11243965

**Published:** 2022-12-07

**Authors:** Haiyan Yang, Yongkang Duan, Zhiwen Wei, Yaqiong Wu, Chunhong Zhang, Wenlong Wu, Lianfei Lyu, Weilin Li

**Affiliations:** 1 Institute of Botany, Jiangsu Province and Chinese Academy of Sciences (Nanjing Botanical Garden Mem. Sun Yat-Sen), Jiangsu Key Laboratory for the Research and Utilization of Plant Resources, Nanjing 210014, China; 2 College of Forestry, Nanjing Forestry University, Nanjing 210037, China

**Keywords:** blueberry, substrate, soilless cultivation, fruit quality, metabolite

## Abstract

With improving living standards, traditional blueberry planting modes cannot meet commercial demands, and blueberry cultivation with soilless substrate has become a popular solution in the blueberry industry. In this study, different soilless substrate treatments were found to markedly influence fruit appearance and intrinsic quality. The fruit in the 50:50 peat/pine bark (*v*/*v*) (FPB) treatment group had the maximum single fruit weight, largest vertical diameter, and brightest color, as well as the highest 1,1-diphenyl-2-picrylhydrazyl (DPPH) value, solid-acid ratio and anthocyanin content. The fruit in the 50:50 pine bark/rice husk (*v*/*v*) (FBR) treatment group had the highest total phenol and flavonoid levels, largest drip loss value, and lowest total pectin content and firmness value. Metabolomic analysis showed that flavonoid, carbohydrate, and carbohydrate conjugate, and amino acid, peptide, and analog levels were significantly different between groups. Fruit in the FPB group had the highest sucrose, D-fructose 1,6-bisphosphate, salidroside, tectorigenin, naringenin chalcone, trifolirhizin, and galangin contents. The increase in the relative expression of phenylalanine (Phe) promoted the synthesis of fruit polyphenols in the FBR group. Our results provide new insights into the effects of different substrates on the quality of blueberries and a reference for the soilless substrate cultivation of blueberries.

## 1. Introduction

The blueberry is an economically important perennial shrub belonging to the genus *Vaccinium* and family *Ericaceae* [1,2]. In addition to containing sugars, organic acids, amino acids, vitamins and minerals necessary for the human body, the blueberry fruit is also rich in polyphenols, anthocyanins, flavonoids, tannins and other biologically important substances [1,3], especially anthocyanins, which enhance vascular elasticity and antioxidants, protect the liver, delay aging and inhibit the proliferation of cancer cells [1,4]. Thus, blueberries have been recognized worldwide as “superfoods”, and for their health-promoting compounds [3].

Fruit quality is the main factor affecting blueberry commodity value [5]. Blueberry fruit quality includes both appearance and intrinsic quality [1]. Appearance quality mainly includes fruit shape, size, weight, color, etc. The intrinsic qualities mainly include sugar content, organic acid content, the sugar-acid ratio, amino acids, anthocyanins, etc. [1,5]. Fruit color is a core indicator of appearance quality, and excellent color is often the most important feature enhancing market competitiveness and attractiveness to consumers [6]. The color change in blueberry fruit is mainly determined by the type and amount of anthocyanins [7]. Anthocyanins not only affect the color of blueberries but are also important active ingredients in blueberry fruits. Anthocyanins are flavonoids and are among the most important water-soluble pigments in nature. Seven anthocyanin species are commonly found in plants, including pelargonidin, delphinidin, peonidin, cyanidin, malvidin, petunidin and primulin [8]. Blueberry anthocyanins mainly include peonidin, petunidin, cyanidin, delphinidin, and malvidin, of which delphinidin, malvidin, and petunidin are the major contributors to the total anthocyanin content [4].

The sugar and acid contents reflect the flavor of the fruit. Generally, the higher the sugar-acid ratio is, the sweeter the fruit [9]. Sugar is not only an important indicator of the sweetness of the fruit but also a basic raw material for the synthesis of organic acids, pigments, aromatic substances and other nutrients [9,10]. Numerous studies have indicated that sugar may play diverse roles in plant life cycles as an important signal [11]. Amino acids are also important nutrients in the fruit, some of which are related to the synthesis of fruit flavor substances [12]. The amino acids involved in the synthesis of flavor substances are called delicious amino acids. According to the human sense of taste, delicious amino acids can be divided into flavor amino acids (glutamic acid and aspartic acid), sweet amino acids (alanine, glycine, serine, threonine, and proline) and bitter amino acids (valine, isoleucine, leucine, arginine, phenylalanine (Phe), histidine, and methionine) [12,13]. Fruit quality is related not only to the genetic factors of the plant but also to external factors such as cultivation medium, light, temperature, and nutrition, which are also the main determinants of fruit quality [10,14]. Therefore, in cultivation and production practices, how to use external environmental factors to regulate the yield of blueberries, promote the synthesis and accumulation of nutrients such as anthocyanins and amino acids in blueberry fruits, and improve the quality of blueberry fruits are focuses of contemporary blueberry research [15].

The blueberry is native to North America and is now widely cultivated in many other countries, such as Poland, Canada, and China [5,15]. In recent years, China’s blueberry cultivation area and yield have risen rapidly, five major blueberry-producing areas have initially formed, and blueberries have become the fastest-growing emerging fruit trees in China [16,17]. Currently, the cultivation of blueberries in China is mainly based on traditional open-field cultivation [17]. The traditional soil cultivation method consumes considerable personpower and material resources in the cultivation process of blueberries, but the harvest yield has not increased significantly, the process is inefficient, and the problems of soil-borne diseases, insect pests, and continuous cropping seriously hinder the development of the blueberry industry [18]. With economic development and the rising cost of labor, the blueberry-growing industry urgently needs a simple cultivation method that can improve the quality of blueberry fruit [15]. In addition, as a functional fruit in China, blueberries have become increasingly demanded by consumers, and their quality directly determines their price and their planting benefits to fruit farmers [16].

With the development of facility agriculture, soilless cultivation technology has received increasing attention [17]. Substrate cultivation can effectively avoid cultivation obstacles, soil-borne diseases, and insect pests, and has the advantages of a controllable production process, high yield, high quality, and reduced pollution [18]. Therefore, to study the effects of different cultivation substrates on the fruit quality of blueberries, screening out cultivation substrates suitable for the growth of blueberries is a very important step in improving the adaptability of blueberries in southern China and improving the quality of the fruits. At present, the commonly used soilless cultivation substrates are mainly grass charcoal, perlite, vermiculite, rock wool, fine sand, etc. A single substrate has difficulty meeting all the physical and chemical environmental requirements for crop growth. As a result, numerous studies on the characteristics of mixed substrates have been conducted, and the results have proven that a mixed substrate with a certain ratio is more conducive to the growth of crops than a single substrate [15,17,18]. According to the results of recent domestic and foreign research, the development and production of substrates have mainly been performed for vegetable cultivation, with only a small part applied to ornamental plants and fruit trees; the research is not sufficiently systematic, and a large number of experimental cultivations are required for substantive and practical exploration [18]. In this study, three substrates, peat, pine bark, and rice hull, were used as raw materials, and the effects of different substrate formulations on the appearance and quality components of blueberry fruit were evaluated. The significantly altered metabolites were investigated by metabolomic technology. Our results extend knowledge of the metabolic basis of important blueberry fruit traits and provide a reference for future research.

## 2. Materials and Methods

### 2.1. Plant Material and Treatment Description

A pot culture experiment was conducted in a greenhouse at the Blueberry Research Base, Modern Agricultural Industrial Park, Baima, Lishui District, Nanjing, China (E119°11′, N31°36′). The blueberry cv. ‘Zhaixuan 7’ (southern highbush) was used as the experimental material. One-year-old seedlings with uniform size were selected and planted into individual 10 L (diameter 28 cm; height 30 cm) pots with different culture substrates. Three substrate formulations were prepared: (1) 50:50 peat/rice husk (*v*/*v*) (FPR); (2) 50:50 peat/pine bark (*v*/*v*) (FPB); and (3) 50:50 pine bark/rice husk (*v*/*v*) (FBR). The primary properties of the three mixed substrates used here have been previously described [17]. The total N, P, and K contents of the substrates were 3.14 mg g^−1^, 2.43 mg g^−1^, and 0.38 mg g^−1^ (FPR); 2.86 mg g^−1^, 0.14 mg g^−1^, and 0.86 mg g^−1^ (FPB) and 1.49 mg g^−1^, 0.28 mg g^−1^, and 3.46 mg g^−1^ (FBR), respectively. Among them, the FPR group was set as the control (CK). The experiment was arranged following a completely randomized design with three replicates per treatment. Plants were irrigated with water to maintain adequate medium moisture and fertigated with nutrient solution every three days to provide essential nutrients. The nutrient solution was a water-soluble fertilizer (Shandong Jining Jinshan Biological Engineering Co., Ltd., Jining, E115°54′, N34°25′, China). Ripe fruits were sampled from each treatment for measurements in June 2022. All of the samples were flash-frozen in liquid nitrogen and kept at −80 °C until further analysis.

### 2.2. Measurement of the Fruit Appearance Index, Firmness and the Diameter of the Stem Scar

The fruit transverse and longitudinal diameters and the stem scar diameter were measured with a digital Vernier caliper. The fruit weight was measured with an electronic balance. The color of the blueberry fruit was measured using a colorimeter (3NH SR-66; Shenzhen 3NH Technology Co., Ltd., Shenzhen, China). The relevant color parameters were brightness (L *), red/green value (a *), and blue/yellow value (b *), and the measurement aperture was 8 mm. The illumination source was an artificial daylight 6500 K (D65), and the sensor was a silicon photodiode. The measurement time was 1.5 s. Fruit firmness was assayed using a fruit firmness meter (Catalog No. 9300 (KM-5), Kyoto, Japan). The probe diameter and downward pressure distance were 5 mm and 10 mm, respectively. The unit was kg cm^−2^.

### 2.3. Determination of Pectin Content and the Drip Loss

The total pectin content was determined by the carbazole colorization method, according to NY/T 2016–2011 (determination of pectin content in fruits and their products). Fresh fruit (1.0 g) was ground to a homogenate and mixed with 35 mL of 70 °C absolute ethanol. Then, the mixture was incubated in a water bath at 85 °C for 10 min. After washing with 67% ethanol, sulfuric acid solution (pH 0.5) was added to the precipitate and kept warm in a water bath at 85 °C for 60 min. The mixture was cooled to room temperature and centrifuged at 8000× *g* for 15 min, and the supernatant was fixed to the appropriate volume and diluted at the appropriate multiple to be measured. Then, 1.0 mL of the extract was added to 0.25 mL of 1 mg mL^−1^ carbazole-ethanol solution and 5 mL of concentrated sulfuric acid, followed by immersion in an 85 °C water bath for 20 min and cooling for 15 min. The absorbance was measured at 525 nm.

The drip loss was measured using the method of Žlabur et al. [19]. Frozen fruits were removed from the refrigerator, weighed immediately and the weights recorded as M1. After thawing completely, the surface juice was dried, weighed, and the weights recorded as M2. The percentage of the reduced mass of the sample and the original mass of the sample was calculated as the drip loss in blueberry fruit after thawing. Drip loss (%) = (M1 − M2)/M1 × 100%.

### 2.4. Determination of Total Flavonoid and Ellagic Acid Contents

The total flavonoid content was measured according to the state standard GBT 20574-2006. Fresh fruit samples (3 g) were extracted in 30 mL of 95% ethanol for 45 min at 65 °C. The suspension was centrifuged at 5000× *g* rpm for 5 min at 4 °C. Then, 3 mL of the supernatant was mixed with 10 mL of 95% ethanol, 1 mL of 100 g L^−1^ Al(NO)_3_, and 1 mL of 9.8 g L^−1^ CH_3_COOK. The mixture was diluted to 50 mL with deionized water and incubated at room temperature for 1 h. The measurements were taken at 415 nm.

The method for determining ellagic acid content was described by Maas et al. [20]. Fresh fruit samples (3 g) were homogenized with 30 mL of 40% ethanol. After ultrasonic surging and incubation in a water bath at 80 °C for 20 min, the extraction was centrifuged at 7000× *g* for 10 min at room temperature. The supernatant (1 mL) was then mixed with 4 mL of 0.1 M NaOH and allowed to rest for 15 min to enable color development, at which point the absorbance was measured at 357 nm.

### 2.5. Determination of Total Phenol, Total Anthocyanin, Soluble Solids, and Total Acid Contents

The total phenol content was detected using the Folin–Ciocalteu method [21]. The total anthocyanin content was measured by the pH differential method [22]. The soluble solid content was obtained using a handheld Brix Meter (Atago, WYT-A, Tokyo, Japan). The total acid content was measured via acid-base titration according to the GB/T12456-2008 determination of total acid in food, using citric acid as a standard. The solidity-acid ratio was calculated according to the ratio between the soluble solid content and the total acid content.

### 2.6. Determination of 1,1-diphenyl-2-picrylhydrazyl (DPPH)s

Half a gram of fresh fruit sample was ground with 4.5 mL of phosphate buffer (0.05 M, pH 7.8). After centrifugation, the supernatant was collected for testing. The fructose and glucose contents were determined according to fructose (A085) and glucose (F006) testing kits (Nanjing Jiancheng Institute of Bioengineering, Nanjing, China). Fructose can be dehydrated under acidic conditions to form 5–hydroxyfuranformaldehyde. To measure the fructose content, 0.05 mL of the supernatant and 3 mL of concentrated hydrochloric acid were blended and incubated in water for 8 min at 100 °C. After cooling, the absorbance was measured at 285 nm. The glucose content was assayed by the glucose oxidase method. The reaction mixture (1 mL) consisted of 5 mM phenol, 0.5 mM 4-aminoantipyrine, ≥15,000 U L^−1^ glucose oxidase, ≥1000 U L^−1^ peroxidase, and the sugar extract. Then, the mixture was kept in water at 37 °C for 15 min to perform the reaction. Finally, the absorbance was measured at 505 nm.

### 2.7. Determination of the Generation Rate of O_2_^·−^, H_2_O_2_ and MDA Contents and Antioxidant Enzyme Activities

The hydroxylamine chloride method was used to determine the generation rate of O_2_^·−^, according to Wang and Luo [23]. The malondialdehyde (MDA) content was measured by the thiobarbituric acid (TBA) method [24]. The H_2_O_2_ content was estimated by an H_2_O_2_ testing kit (Nanjing Jiancheng Bioengineering Institute, Nanjing, China). Superoxide dismutase (SOD) activity was assessed by the nitro blue tetrazolium (NBT) method [25]. Peroxidase (POD) activity was measured as the oxidation rate of guaiacol [26]. Catalase (CAT) activity was measured by the ammonium molybdate method [27].

### 2.8. Determination of FRAP and DPPH Radical Scavenging Capacity

The ferric reducing antioxidant power (FRAP) and 1,1-diphenyl-2-picrylhydrazyl (DPPH) radical scavenging capacity were estimated by FRAP and DPPH testing kits (Nanjing Jiancheng Bioengineering Institute, Nanjing, China). The results were expressed as the amount of the antioxidant Trolox. For FRAP, under acidic conditions, the antioxidants can reduce Fe^3+^-TPTZ to produce blue Fe^2+^-TPTZ, which has strong absorption at 593 nm. The final result was expressed as the concentration of the FeSO_4_ standard solution. DPPH radicals have single electrons, and their alcohol solution is purple and has strong absorption at 517 nm.

### 2.9. Metabolomic Analysis

#### 2.9.1. Metabolite Extraction

A fruit sample (0.1 g) was ground into powder using liquid nitrogen and extracted in an ice bath for 5 min with 500 μL of 80% aqueous methanol solution. After centrifugation at 15,000× *g* and 4 °C for 20 min, the extracts were added to mass spectral grade water and diluted to a methanol content of 53%. Following centrifugation at 15,000× *g* and 4 °C for 20 min, the extracts were filtered using a 0.22 μm pore size and finally transferred to sample vials for metabolomic analysis [28].

#### 2.9.2. UHPLC—MS/MS Analysis

UHPLC—MS/MS analyses were performed using a Vanquish UHPLC system (Thermo Fisher, Hannover, Germany) coupled with an Orbitrap Q Exactive^TM^ HF mass spectrometer (Thermo Fisher, Hannover, Germany) according to the manufacturer’s guidelines. The system was equipped with a Hypersil Gold column (100 mm × 2.1 mm, 1.9 μm) operating at 40 °C and a flow rate of 0.2 mL min^−1^. In positive ionization mode, eluent A was 0.1% formic acid, and eluent B was methanol. In negative ionization mode, eluent A and eluent B were 5 mM ammonium acetate and methanol, respectively. The solvent gradient is shown in Table 1, and the mass spectrometric parameters are shown in Table 2.

#### 2.9.3. Data Processing and Metabolite Identification

The mass spectrometry raw data were processed with Compound Discoverer 3.1 (CD3.1, Thermo Fisher) for peak alignment, peak selection, and quantitation of each metabolite. After normalizing the peak intensities to the total spectral intensity, the metabolite peaks were matched with the mzCloud (https://www.mzcloud.org/, accessed on 12 September 2022), mzVault and MassList databases. The annotation of metabolites was performed using the Human Metabolome (HMDB) database and the Lipid Maps database.

### 2.10. Statistical Analysis

Principal component analysis (PCA) and partial least squares discriminant analysis (PLS-DA) were carried out to examine the separation of individual samples and the overall difference in metabolites between the two groups. Statistical significance (*p* value) was determined by univariate analysis (t test). The screening criteria for the differentially abundant metabolites (DAMs) were variable importance in projection (VIP) value > 1, *p* value < 0.05 and fold change (FC) ≥ 2 or FC ≤ 0.5 [29,30]. The functions and metabolic pathways of the DAMs were analyzed using the Kyoto Encyclopedia of Genes and Genomes (KEGG) database. The metabolic pathways were considered enriched when the ratio satisfied x/n > y/n and were statistically significant when the *p* value < 0.05. The fruit physiological and quality index data were all analyzed with SPSS 20.0 software and Microsoft Office Excel 2016. One-way analysis of variance (ANOVA) and Tukey’s test were performed to detect possible differences among the different substrate treatments, and significant differences were expressed as *p* < 0.05. Correlation analysis and PCA of physiological and quality indexes were performed with Origin 2022 (Origin Lab Inc., Northampton, MA, USA).

## 3. Results

### 3.1. Fruit Weight, Size and Color

As Table 3 shows, substrate type had a significant impact on fruit appearance. The fruits of the FPB treatment group were the largest, and the single-fruit weight, transverse diameter and vertical diameter values were the largest. The single-fruit weight was significantly different from that in the FBR treatment group and FPR (CK) group. The vertical diameter in the FPB and FBR treatments differed significantly from that in the FPR (CK) group. There were also certain differences in fruit color under different substrate treatments (Table 3). L * indicates the brightness of the fruit: negative values represent darkness and positive values represent lightness. a * represents the color value from green (negative values) to red (positive values), and b * represents the value from blue (negative values) to yellow (positive values). The brightness of the fruit in the FPB treatment group was the highest. The color of the fruit in the FBR group was closer to red than that in the other two groups. The color of the fruit in the FPB treatment group was closer to blue. The comprehensive analysis suggested that the color performance of the FPB treatment group was the best.

### 3.2. Fruit Firmness, Pectin Content, the Size of the Stem Scar, and the Drip Loss

The firmness of the fruit affects its storage and taste. As shown in Table 4, the fruit firmness in the FPB and FBR treatment groups was 8.86% and 10.33% lower than that in the FPR (CK) group, respectively. Pectin plays an important role in the biomechanics of the cell wall and directly promotes the cross-linking of cellulose microfibers in the cell wall. The change in the pectin structure leads to loosening and disorganization of the cell wall, which is an important cause of fruit ripening and softening. The total pectin content of the fruit in the FBR treatment group was 17.12% lower than that in the FPR (CK) group (Table 4). The stem scar is a small wound caused by the rupture of the pericarp and damage to the pulp tissue when the stalk is separated from the fruit. It is usually the key point for the fruit to be infected by mold and to lose water. The larger the stem scar, the more likely the fruit is to lose water, shrink, and become moldy. In this study, the stem scar size is indicated by the diameter of the stem scar. However, there was no significant difference in the diameter of the stem scar between different treatments (Table 4). The drip loss is an important index to measure fruit quality during the frozen storage period, which can represent the water holding capacity of frozen samples, and reflect the flavor and nutrient preservation of frozen samples. Compared with the control (FPR), the drip loss of blueberry fruit in FBR treatment was significantly increased, but there was no significant difference between the FPB treatment group and the control group (Table 4). It can be seen that the nutrients of blueberry fruits in FPB and CK groups were better preserved, which indicated that the shelf-life of the fruits cultivated in these two substrates was higher to a certain extent.

### 3.3. Fruit Antioxidant System Indexes

**Figure 1:**
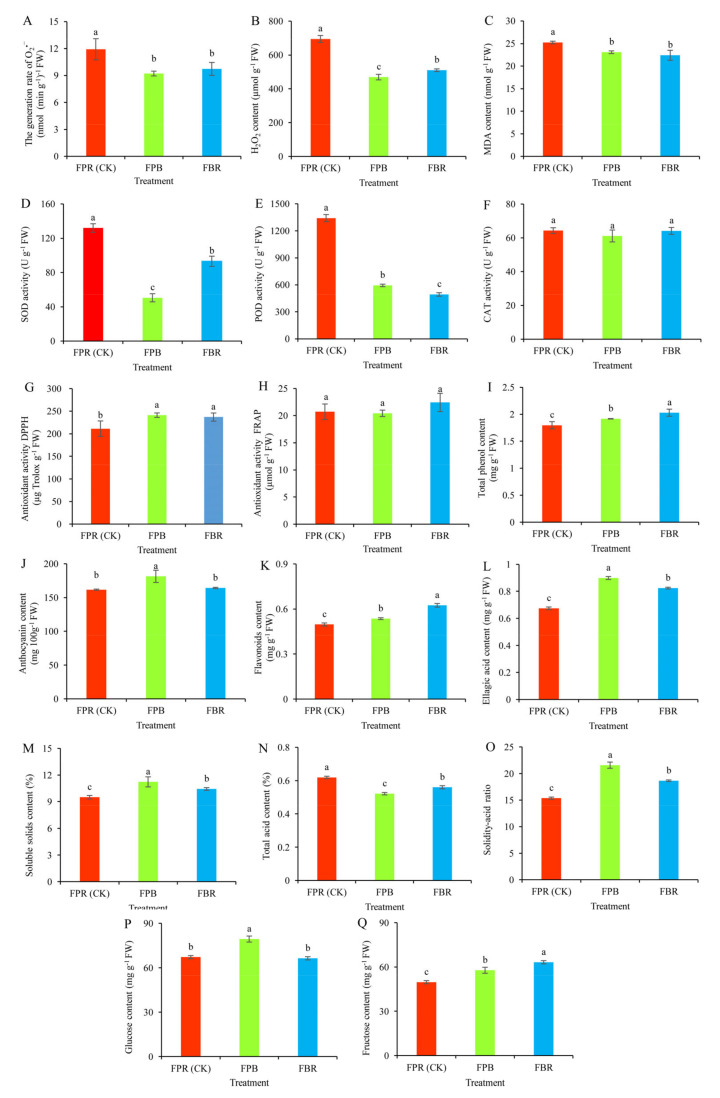
Effects of the soilless substrate type on the antioxidant system and quality indexes of blueberry fruits. (**A**) The generation rate of O_2_^−^; (**B**) H_2_O_2_ content; (**C**) MDA content; (**D**) SOD activity; (**E**) POD activity; (**F**) CAT activity; (**G**) Antioxidant activity DPPH, (**H**) Antioxidant activity FRAP; (**I**) Total phenol content; (**J**) Anthocyanin content; (**K**) Flvaonoids content; (**L**) Ellagic acid content; (**M**) Soluble solids content; (**N**) Total acid content; (**O**) Solidity-acid ratio; (**P**) Glucose content; and (**Q**) Fructose content. Values are presented as the mean ± SD (*n* = 3). Bars with different letters indicate significant differences between the treatments according to Tukey’s tests (*p* < 0.05).

Superoxide anion radicals (O_2_^−^) and H_2_O_2_ are the main free radicals generated during plant metabolism. Under FPR (CK) substrate treatment, the generation rate of O_2_^−^ and H_2_O_2_ increased significantly (Figure 1A,B). As an indicator of plant oxidative damage, the MDA content in the FPR (CK) group significantly increased by 10.02% and 17.11% compared with that in the FPB and FBR treatment groups, respectively (Figure 1C). The activities of two important protective enzymes of the antioxidant system, SOD and POD, significantly increased in the FPR (CK) group. Compared with that in the FPB and FBR treatment groups, SOD activity was increased by 161.47% and 41.61%, respectively, in the FPR (CK) group (Figure 1D). Similarly, POD activity was increased by 126.16% and 172.18%, respectively (Figure 1E). CAT activity did not differ significantly among the three treatments (Figure 1F). DPPH free radical scavenging capacity of the fruit in the FPB and FBR treatments was significantly higher than that in the FPR (CK) substrate treatment (Figure 1G). However, the FRAP values of the fruit did not differ significantly between the different substrates (Figure 1H).

### 3.4. Fruit Quality Indexes

Total phenol, anthocyanin, flavonoid, and ellagic acid contents are important determinations of active substances in blueberry fruit. Under different substrate treatments, the total phenol content decreased in the order of the FBR group, FPB group, and FPR (CK) group, and the difference in contents among the three was significant (Figure 1I). In the FPB treatment group, the anthocyanin content was the highest and was 12.29% and 10.52% higher than that in the FPR and FBR treatment groups, respectively (Figure 1J). In terms of the flavonoid content from high to low, the treatment groups were ordered FBR, FPB and FPR (CK), and the difference among the three groups was significant (Figure 1K). The content of ellagic acid in the FPB treatment group was significantly increased by 33.24% and 9.08% compared with that in the FPR and FBR treatment groups, respectively (Figure 1L). Soluble solid and total acid contents are common indicators of blueberry fruit quality, and the solidity-acid ratio is closely related to fruit flavor. Under FPB substrate treatment, the solid content and solid-acid ratio were significantly increased compared with those in the FPR (CK) and FBR treatment groups (Figure 1M,O). The total acid content in the FPB treatment group was the lowest, indicating that the fruit flavor of the FPB treatment group was much better (Figure 1N). In addition, in the FPB treatment group, the glucose content was the highest (Figure 1P). Compared with the FPR (CK) group, the FPB treatment group also had a high fructose content (Figure 1Q).

### 3.5. Correlation and PCA of Physiological and Quality Indexes

**Figure 2:**
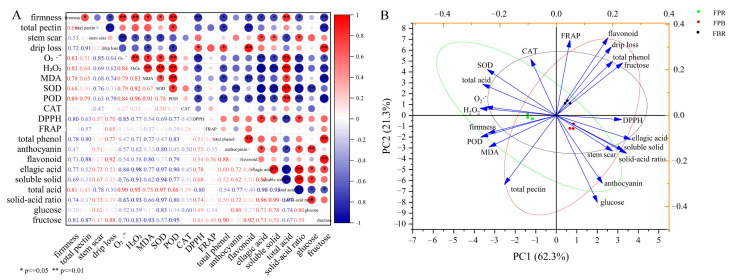
(**A**) Correlation matrix of the physiological and quality indicators of blueberry fruits. The results were derived from the Pearson correlation analysis. Red and blue colors indicate positive and negative correlations, respectively. * and ** represent a significant correlation at the 0.05 and 0.01 levels, respectively. (**B**) PCA score plot of the physiological and quality indicators of blueberry under different substrate treatments.

As shown in Figure 2A, the fruit firmness was significantly positively correlated with the total pectin and MDA contents and SOD activity (*p* < 0.05), as well as the generation rate of O_2_^·−^, H_2_O_2_ and total acid contents, and POD activity (*p* < 0.01), whereas fruit firmness was negatively correlated with the values of the drip loss, DPPH and solid-acid ratio, and the contents of total phenol, flavonoid, ellagic acid, soluble solid, and fructose (*p* <0.05). The DPPH value and the contents of the total phenol, flavonoid and fructose were positively correlated with the drip loss (*p* < 0.05). Moreover, the generation rate of O_2_^·−^, the contents of H_2_O_2_ and MDA, and the activities of SOD and POD were negatively correlated with the values of the DPPH and the solid-acid ratio, and the contents of total phenol, flavonoid, ellagic acid and fructose, but positively correlated with the total acid content (*p* < 0.05). The anthocyanin content was positively correlated with the contents of ellagic acid and glucose, and the solid-acid ratio (*p* < 0.05).

Principal component analysis (PCA) showed that the contribution rate of the two principal components (PC1 and PC2) was 62.3% and 21.3%, respectively, and the cumulative variance contribution rate was 83.6% (>75%). Therefore, the two principal components accurately covered the information of 21 indicators. The PCA results also showed that the scattered points corresponding to each treatment were clearly separated, suggesting that different substrate treatments had significant effects on various indexes of the blueberry (Figure 2B). Moreover, fructose, DPPH, ellagic acid, soluble solid, and solid-acid ratio showed a positive correlation with PC1, whereas total acid content, firmness, O_2_^·−^, H_2_O_2_, MDA, SOD, and POD had a negative correlation with PC1. SOD, CAT, FRAP, flavonoid, drip loss, total phenol, and fructose were positively correlated with PC2, whereas soluble solid, solid-acid ratio, stem scar, total pectin, anthocyanin, and glucose were negatively correlated (Figure 2B).

### 3.6. Metabolite Profiling and Classification

To investigate the differences in metabolite accumulation of blueberry fruits grown in different soilless substrates, a total of 839 metabolites were quantified in nontargeted metabolomic analysis of blueberry fruits using LC—MS, of which 479 metabolites were in positive ionization mode and 360 metabolites were in negative ionization mode (Table S1). The KEGG, HMDB, and Lipid Maps databases were used for the functional and categorical annotation of the identified metabolites. KEGG annotation revealed 185 and 175 metabolites in positive and negative ionization modes, respectively, with metabolism concentrated in global and overview maps, amino acid metabolism, biosynthesis of other secondary metabolite pathways, carbohydrate metabolism, and metabolism of cofactors and vitamin pathways (Figure S1A,B). HMDB annotation showed that the metabolites were mainly phenylpropanoids, polyketides, lipids, and lipid-like molecules (Figure S1C,D). Lipid Maps annotation revealed that the metabolites were mainly flavonoids (Figure S1E,F).

### 3.7. Multivariate Statistical Analysis

**Figure 3:**
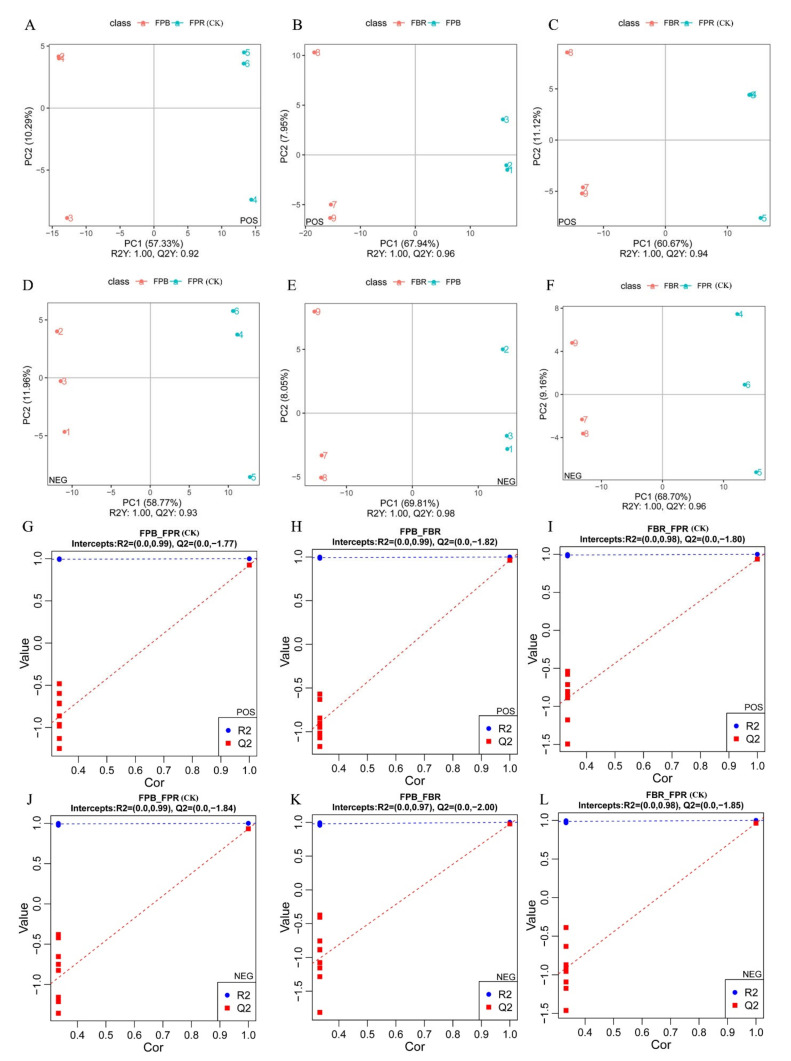
Partial least squares discrimination analysis (PLS-DA) of metabolites in blueberry fruits under three different soilless substrate treatments. PLS-DA score plot analysis of the three comparisons in positive (FPB vs. FPR (CK) (**A**), FPB vs. FBR (**B**), and FBR vs. FPR (CK) (**C**)) and negative (FPB vs. FPR (CK) (**D**), FPB vs. FBR (**E**), and FBR vs. FPR (CK) (**F**)) ionization modes. PLS-DA valid plot analysis of the three comparisons in positive (FPB vs. FPR (CK) (**G**), FPB vs. FBR (**H**), and FBR vs. FPR (CK) (**I**)) and negative (FPB vs. FPR (CK) (**J**), FPB vs. FBR (**K**), and FBR vs. FPR (CK) (**L**)) ionization modes.

PCA mainly reflects the characteristics of original datasets by determining the degree of dispersion between the components in a sample, and principal component 1 (PC1) represents the most obvious feature in the multidimensional data matrix. As shown in the PCA score plot, the variance contribution of PC1 to the three comparisons of FPB vs. FPR (CK), FPB vs. FBR, and FBR vs. FPR (CK) in positive ion mode was 54.89%, 63.28%, and 56.23%, respectively, and that of the second principal component (PC2) was 13.08%, 10.42%, and 13.74%, respectively (Figure S2A–C). In negative ionization mode, the PC1 values for the three comparisons were 54.60%, 65.21%, and 64.45%, and the PC2 values were 14.45%, 11.03%, and 10.99% (Figure S2D–F). There were obvious differences between the metabolites of blueberry fruits under different substrate treatments, and the differences in metabolites within the groups were smaller than those between groups. The accuracy and reliability of the data were further evaluated by building a PLS-DA model (Figure 3). As shown in the PLS-DA model, the R2Y values of the different groups were all 1.00, and the R2Y values were greater than the Q2Y values, suggesting that the model was well established and that its prediction ability was excellent. Then, an overfit test was conducted to examine the quality of the model. The R2 value was greater than the Q2 value, and the Y-intercept of the regression line of Q2 was less than 0, implying that the model was not overfitted.

### 3.8. DAM Identification and Analysis

**Figure 4:**
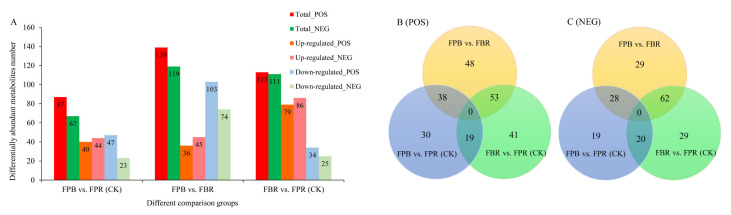
Differentially abundant metabolites (DAMs) in blueberry fruits identified in the three comparisons (FPB vs. FPR (CK), FPB vs. FBR, and FBR vs. FPR (CK)). (**A**): Histogram showing the number of DAMs, including the total number, upregulated number, and downregulated number in positive and negative ionization modes. (**B**,**C**): Venn diagrams of DAMs in positive and negative ionization modes, respectively.

The screening of DAMs mainly includes VIP, FC and *p* values, and the thresholds are VIP > 1.0, FC > 1.5 or FC < 0.667 and *p* value < 0.05. In positive ionization mode, 139, 87, and 113 DAMs were screened in the FPB vs. FBR, FPB vs. FPR (CK), and FBR vs. FPR (CK) comparisons, respectively. In the three comparisons, 36, 40, and 79 DAMs were upregulated and 103, 47 and 34 DAMs were downregulated (Figure 4A), respectively. In negative ionization mode, 119, 67, and 111 DAMs were screened in the FPB vs. FBR, FPB vs. FPR (CK), and FBR vs. FPR (CK) comparisons, respectively. In these three comparisons, 45, 44, and 86 DAMs increased in expression level, while 74, 23, and 25 DAMs decreased in expression level (Figure 4A). To further compare the differences in DAMs, a Venn diagram was generated, and the results showed that in positive ionization mode, 38, 19, and 53 DAMs were common in the FPB vs. FBR and FPB vs. FPR(CK) comparisons, FPB vs. FPR (CK) and FBR vs. FPR (CK) comparisons, and FPB vs. FBR and FBR vs. FPR (CK) comparisons, respectively (Figure 4B). In negative ionization mode, 28, 29, and 62 DAMs were common in the FPB vs. FBR and FPB vs. FPR (CK), FPB vs. FPR (CK), and FBR vs. FPR (CK), and FPB vs. FBR and FBR vs. FPR (CK) comparisons, respectively (Figure 4C).

**Figure 5:**
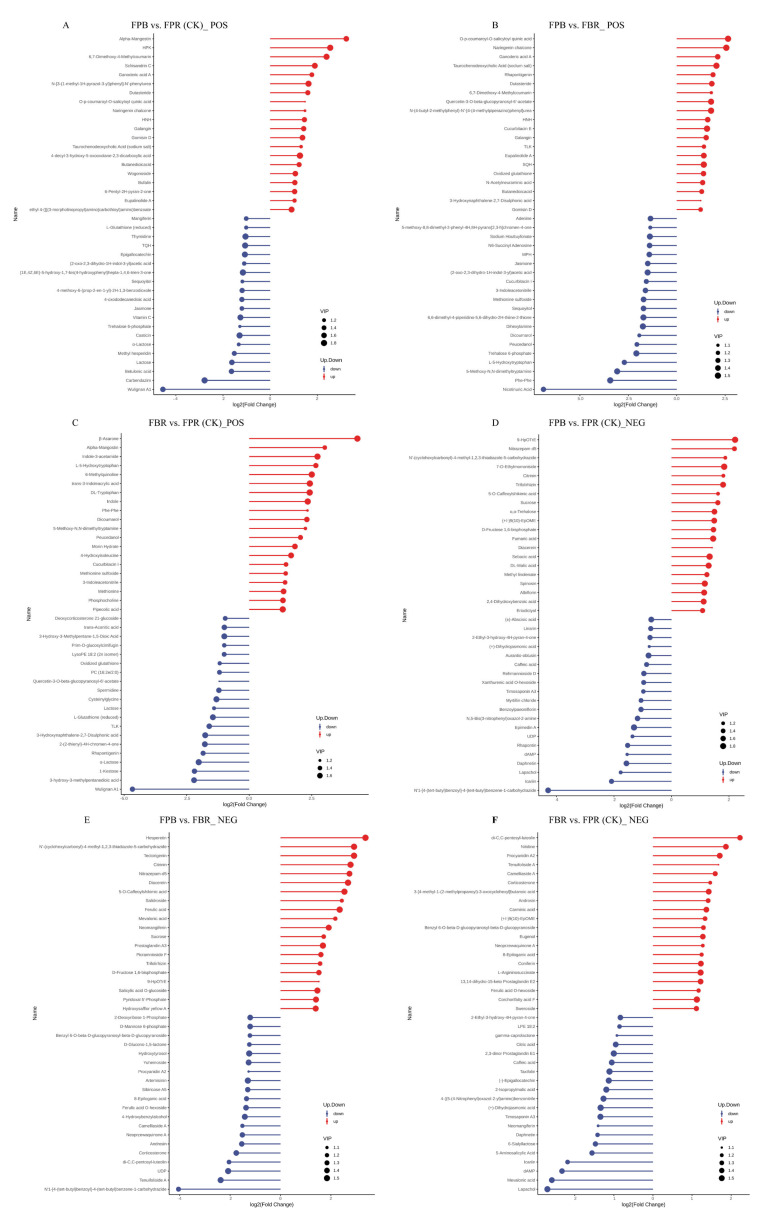
Stem diagram of the top 20 upregulated and downregulated differentially abundant metabolites (DAMs) identified from the three comparisons in positive (FPB vs. FPR (CK) (**A**), FPB vs. FBR (**B**), and FBR vs. FPR (CK) (**C**)) and negative (FPB vs. FPR (CK) (**D**), FPB vs. FBR (**E**), and FBR vs. FPR (CK) (**F**)) ionization modes. Red dots represent upregulated DAMs, and blue dots represent downregulated DAMs. The length of the stem represents the value of log2 (fold change), and the size of the dot represents the VIP value.

With further analysis of the top 20 upregulated and downregulated DAMs, a stem diagram was obtained (Figure 5). The results showed that in positive ionization mode (Figure 5A–C), the expression level of 11 identical DAMs increased, and the expression level of 4 identical DAMs decreased in the two comparisons of FPB vs. FPR (CK) and FPB vs. FBR. Specifically, naringenin chalcone (chalcone and dihydrochalcone subclass) was increased 1.5-fold and 2.6-fold, galangin (flavone subclass) was increased 1.4-fold and 1.5-fold, and trehalose 6-phosphate (carbohydrates and carbohydrate conjugates subclass) was downregulated 1.3-fold and 2.1-fold, respectively. In the FPB vs. FPR (CK) and FBR vs. FPR (CK) comparisons, four identical DAMs were significantly downregulated. Lactose (carbohydrates and carbohydrate conjugates subclass) was downregulated 1.6-fold and 1.4-fold, respectively. In negative ionization mode (Figure 5D–F), 9 identical DAMs were significantly accumulated, while 2 identical DAMs were significantly reduced in the FPB vs. FPR (CK) and FPB vs. FBR comparisons. Specifically, the expression level of trifolirhizin (furanoisoflavonoid subclass) was increased 1.8-fold and 1.6-fold, respectively, and the expression level of sucrose (carbohydrates and carbohydrate conjugates subclass) was increased by 1.6-fold and 1.7-fold, respectively. The fruit D-fructose 1,6-bisphosphate (carbohydrates and carbohydrate conjugates subclass) was significantly elevated by 1.5-fold. Moreover, in the FPB vs. FPR (CK) and FBR vs. FPR (CK) comparisons, one DAM was significantly increased, and 8 identical DAMs were significantly reduced, of which icariin (flavone and flavonol subclasses) was markedly downregulated 2.1-fold and 2.2-fold, respectively.

### 3.9. Correlation Analysis of DAMs

The consistency of changes in metabolites was performed by analyzing the correlations between various metabolites. Overall, with the exception of the FPB vs. FBR comparison in negative ionization mode, the number of positively correlated DAMs in the remaining comparisons was greater than the number of negatively correlated DAMs (Figure S3). In addition, in positive ionization mode, there was a significant positive correlation between the top 10 DAMs in the FPB vs. FPR (CK) comparison. A significant positive correlation between the top 4 DAMs in the FPB vs. FBR comparison and the top 7 DAMs in the FBR vs. FPR (CK) comparison was also observed. In negative ionization mode, there was a significant positive correlation between the top 7 DAMs in the FPB vs. FPR (CK) comparison. Correlation analysis showed that there was a synergistic or mutually exclusive relationship between the fruit DAMs under different substrate treatments.

### 3.10. KEGG Enrichment Analysis of DAMs

**Figure 6:**
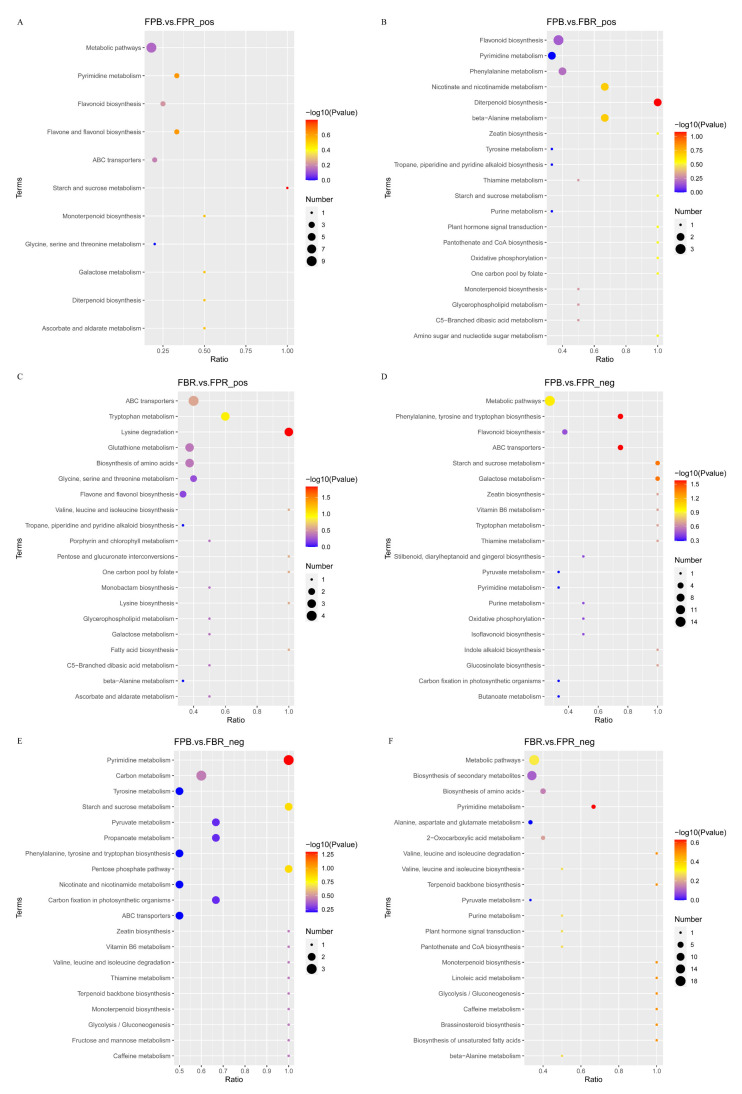
Top 20 enriched KEGG pathways of differentially accumulated metabolites (DAMs) identified from the three comparisons in positive (FPB vs. FPR (CK) (**A**), FPB vs. FBR (**B**), and FBR vs. FPR (CK) (**C**)) and negative (FPB vs. FPR (CK) (**D**), FPB vs. FBR (**E**), and FBR vs. FPR (CK) (**F**)) ionization modes.

To further understand the changes in DAMs in metabolic pathways, the KEGG database was used to annotate the DAMs and perform pathway enrichment analysis. The top 20 metabolic pathways that were significantly enriched were selected for bubble diagram display (Figure 6). The results showed that in positive ionization mode, metabolic pathways were the most concentrated pathways in the FPB vs. FPR (CK) comparison, followed by the pyrimidine metabolism and flavonoid biosynthesis pathways. The most characterized pathways in the FPB vs. FBR comparison included flavonoid biosynthesis, pyrimidine metabolism, and Phe metabolism pathways. In the FBR vs. FPR (CK) comparison, ABC transporters, tryptophan metabolism, and lysine biosynthesis pathways were associated with the most DAMs. In negative ionization mode, in the FPB vs. FPR (CK) comparison, the most characterized pathways included metabolic pathways, Phe, tyrosine and tryptophan biosynthesis, and flavonoid biosynthesis pathways. In the FPB vs. FBR comparison, pyrimidine metabolism, carbon metabolism, and tyrosine metabolism were most enriched. In the FBR vs. FPR (CK) comparison, metabolic pathways, biosynthesis of secondary metabolites, and biosynthesis of amino acids pathways were associated with the most DAMs. Different cultivation substrate treatments had marked influences on the metabolism of flavonoids, carbohydrates, and amino acids in the fruit. In addition, it was also found that the FPB vs. FBR and FBR vs. FPR (CK) comparisons yielded more enriched pathways and DAMs than the FPB vs. FPR (CK) comparison.

### 3.11. Flavonoids, Carbohydrates, and Amino Acids in Blueberry Fruits

**Figure 7:**
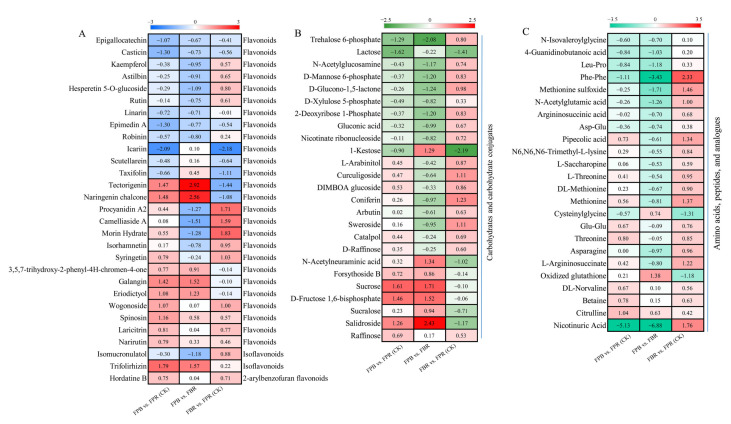
Heatmap of the differentially accumulated metabolites (DAMs) involved in flavonoid (**A**), carbohydrate and carbohydrate conjugate (**B**) and amino acid, peptide, and analog (**C**) metabolism in blueberry fruits overlapping among the three comparisons. The scales were generated using log2 (fold change) values.

As shown in Figure 7, a total of 29 differentially accumulated flavonoid metabolites, 25 differentially accumulated carbohydrate metabolites, and 24 differentially accumulated amino acid metabolites were identified in the three comparisons. Flavonoid-related metabolites belong to the classes of flavonoids, isoflavonoids, and 2-arylbenzofuran flavonoids. In the FPB vs. FPR (CK), FPB vs. FBR, and FBR vs. FPR (CK) comparisons, 16, 14, and 17 flavonoids accumulated, and 13, 15, and 12 flavonoids declined, respectively (Figure 7A). Among them, tectorigenin and naringenin chalcone were significantly upregulated in the FPB vs. FBR comparison, while icariin was markedly decreased in the FPB vs. FPR (CK) and FBR vs. FPR (CK) comparisons (Figure 7A). Carbohydrates and carbohydrate conjugates and amino acids, peptides, and analogs showed similar changes in the three comparisons. Fifteen and 17 carbohydrates and carbohydrate conjugates were upregulated in the FPB vs. FPR (CK) and FBR vs. FPR (CK) comparisons, respectively, and 16 carbohydrates and carbohydrate conjugates downregulated in the FPB vs. FBR comparison (Figure 7B). Salidroside, D-fructose 1,6-bisphosphate, sucrose, and trehalose 6-phosphate differed most significantly in the FPB vs. FPR (CK) and FPB vs. FBR comparisons (Figure 7B), suggesting that these metabolites played vital roles in regulating fruit flavor. Among the amino acid-related metabolites, 22 amino acids, peptides, and analogs were upregulated in the FBR vs. FPR (CK) comparison, of which the Phe-Phe difference was the most significant. Fifteen amino acids, peptides, and analogs were significantly upregulated in the FPB vs. FPR (CK) comparison (Figure 7C). However, in the comparison of FPB vs. FBR, 19 amino acids, peptides, and analogs were significantly downregulated (Figure 7C). Notably, nicotinuric acid differed significantly in the two comparisons of FPB vs. FPR (CK) and FPB vs. FBR, where it was downregulated 5.13-fold and 6.88-fold, respectively (Figure 7C).

## 4. Discussion

With the development of ecological agriculture and sustainable circular agriculture, soilless substrate cultivation has shown superiority in overcoming soil-borne diseases, soil continuous cropping obstacles, and soil salinization, reducing the amount of pesticides and fertilizers, saving water, etc. [18]. Soilless cultivation has attracted increasing attention, and market demand is increasing [31]. The most important component of soilless substrate cultivation is an appropriate substrate, and the selection of the substrate is critical to the success of cultivation [32]. The function of the substrate is to fix the plant and create good growth conditions for the plant, and the cultivation substrate is the most important element controlling the quality of the rhizosphere environment of the plant [17]. Thus, an appropriate substrate type and formula ratio are critical for the growth of the plant. To solve problems such as soil obstacles in the cultivation of blueberries in facilities and meet the commercial demand for high-quality and safe blueberries, this study replaced traditional soil cultivation with substrate cultivation. The effects of different organic substrate ratios on the appearance and intrinsic quality of blueberry fruits were investigated, which could provide a theoretical basis and production guidance for the substrate cultivation technology of blueberries.

Studies have shown that cultivation using different substrates can have different effects on the growth and development of plants [31,32,33,34]. In a field experiment, four mixed soilless substrates were used for strawberry cultivation, and the results showed that strawberry plants grew most vigorously in 100% coconut coir, and the fruit yield was not significantly different from that under conventional soil cultivation [33]. The initial vegetative growth, fruit yield, and fruit weight of persimmon trees were greatly enhanced when the trees were grown in a coconut coir substrate [32]. Ortiz-Delvasto et al. reported that the blueberry cultivar “Legacy” has better growth and yield after planting in a pure coconut coir substrate compared to a mixed substrate of coconut coir and peat (3:1, *v*/*v*), and the fruit diameter was also relatively large in the former substrate [31]. In this study, the fruit weight in the FPB treatment group was greater than that in the other two groups, and the transverse and vertical diameters of the fruit were also the largest, indicating that the fruit grown in the substrate of peat and bark (1:1, *v*/*v*) was well developed. This result may be due to the vigorous growth of the plant at the vegetative stage, which further promoted fruit development [17]. The higher N content in the FPB substrate may have promoted fruit development. Fallah et al. [34] found that the fruit dimensions and weight of cherry tomatoes increased with increasing N content in the nutrient solution. In addition, the color of the fruit also showed significant differences. The fruit in the FPB treatment group was the brightest, with a blueish color. Since the color of blueberries is mainly related to the content and type of anthocyanins [7], the significantly higher anthocyanin content in the FPB treatment group should be the main reason for the blueish color of the fruit. It was reported that anthocyanin accumulation in plants is most commonly associated with P deficiency [35]. The P content in the FPB group was the lowest among the three substrates, which may partially promote the accumulation of anthocyanins in the fruit. Anthocyanins not only affect color but also have high nutritional value, as well as having antioxidant, anticancer, anti-inflammatory, and other functions [1,4]. It is clear that the cultivation substrate has a significant impact on both the appearance and the intrinsic quality of the fruit. The analyses of Wysocki et al. also indicated that the cultivation substrate has a direct impact on the quality of the fruit, and a peat-coconut (50:50, *v*/*v*) substrate yielded higher sugar, acid, polyphenol, and anthocyanin contents of strawberry fruit [36].

The content of sugar and acid is an important indicator of the flavor quality of blueberries, and it is also an important criterion used by consumers to judge the quality of blueberries [9]. Overall, a relatively high solidity-acid ratio will make the fruit more popular with consumers [10]. The sugar content depends on the synthesis, transport, and metabolism of plant photosynthetic substances [2,37]. In this study, the contents of soluble solids and glucose and the solidity-acid ratio in the fruit of the FPB group were the highest, which was associated with a high chlorophyll content in plant leaves in a previous study [17]. Within a certain range, there is a positive correlation between the chlorophyll content and net photosynthetic rate [37]. This also indicates that the levels of mineral elements in the FPB substrate, especially the N and K levels, may be in a relatively moderate concentration range, which can promote plant growth and facilitate improvements in fruit quality. The total soluble solids content of fruit can be improved by the treatment with appropriate an N/K ratio [34]. Polyphenols are important secondary metabolites in blueberries that can protect plants from a variety of biological and abiotic stresses [1,38]. Previous studies suggested that polyphenols in blueberry fruit mainly include anthocyanins, proanthocyanidins, flavonols, ellagic acid, and catechins [39,40]. This study showed that the total phenol and flavonoid contents were the highest in the FBR treatment group, while the anthocyanin and ellagic acid contents were the highest in the FPB treatment group, indicating that the blueberry fruits from these two treatment groups were rich in polyphenols and had strong antioxidant capacities. Previous studies have shown that a sufficient K content can increase the fruit sugar content, phenolic composition, and antioxidant capacity [41]. The higher K content in the FBR substrate may have increased the accumulation of total phenols and flavonoids in the fruit.

Fruit firmness is also an important evaluation indicator of fruit quality. Changes in fruit firmness will directly affect the taste, commercial value, shelf life, and mechanical damage of the fruit [42]. Previous studies have shown that the higher the firmness of blueberries, the longer the shelf life and the lower the decay rate [43], indicating that the firmness is closely related to the shelf-life of the fruit. In this study, the fruit firmness of the FPR (CK) group was higher than that of the FPB and FBR treatment groups, indicating that the 50:50 peat/rice husk substrate was beneficial for the formation of fruit firmness, and the shelf-life of the fruit in the FPR (CK) group was higher. Previous studies have shown that pectin is a determinant of the mechanical properties of the cell wall [44]. Pectin can control the properties of water at the local level, thereby affecting molecular interactions [45]. The degradation of pectin will cause the separation of pulp cells and lead to a decrease in hardness [46]. In this study, the total pectin content in the fruit of the FPR (CK) group was significantly higher than that in fruit of the FBR treatment group, indicating that pectin, as an important substance connecting each component of the cell wall, may play an important role in the firmness of blueberry fruit. Li et al. reported that a small number of extracellular crystals form during the freezing of the sample, which can damage the cell membrane, resulting in the drip loss during thawing [47]. Thus, the drip loss is related to the formation of ice crystals in fruit, which can reflect the quality of frozen fruit to a certain extent. In this study, the drip loss of FPB and FPR (CK) fruits was lower, indicating that blueberry fruits cultivated in these two substrates had higher self-life. Correlation analysis also showed that the fruit hardness was significantly positively correlated with the total pectin, whereas negatively correlated with the value of the drip loss, indicating that these three indexes were closely related to the self-life of fruit. Fruits with a higher self-life may have a longer shelf life, but this does not mean that the fruit taste is good. In this study, although the fruits in FPR (CK) group had a higher self-life, the content of other quality indexes in the fruit was lower, such as anthocyanins, flavonoids and solids.

Reactive oxygen species (ROS), as a common product of plants exposed to stress, not only cause irreversible damage to cells but also attack macromolecules [48]. Excess ROS accumulation promotes pulp decomposition and reduces fruit quality [49]. Under normal circumstances, the antioxidant system maintains ROS production and metabolism in a dynamic balance. When the plant is in an unsuitable environment, this balance will be affected and disrupted [50]. In this study, the significantly increasing generation rate of O_2_^·−^ and H_2_O_2_ in the FPR (CK) treatment group indicated that the environment may have been unsuitable for the plants. Thus, the 50:50 peat/rice husk substrate may not be appropriate for the development of blueberry fruits. Chrysargyris et al. found that the addition of paper waste into the mixed substrate of olive-stone waste and peat inhibited plant growth and resulted in the overproduction of H_2_O_2_ [51]. Previous studies in the vegetative growth stage of the blueberry have shown that the generation rate of O_2_^·−^ and H_2_O_2_ content in leaves in the PR and BR treatment groups was much higher than that in the PB treatment group [17]. However, the decrease in free radical levels in the fruit of the FBR group may be related to higher contents of total phenols and flavonoids. MDA, a cell membrane lipid peroxidation product, can destroy membrane structure and function and affect the material metabolism of cells [50]. The increased MDA content in the fruit of the FPR (CK) group could have been caused by too many superoxide anions initiating membrane lipid peroxidation. SOD catalyzes O_2_^−^ in plants to produce H_2_O_2_ and O_2_. The higher SOD activity in the FPR (CK) group than in the other groups enhanced the fruit’s ability to remove O_2_^−^. The enzymatic reaction of SOD led to rapid accumulation of H_2_O_2_, which in turn promoted the scavenging action of POD and CAT [49,50]. Compared with that in the FPR (CK) group, the POD activity in the FPB and FBR treatment groups was markedly decreased. At this point, POD played a key role in clearing H_2_O_2_. The study showed that the fruit antioxidant system of the FPR (CK) group was more active, and the plant may be in a stressed condition. There is high competition between vegetative growth and fruit development. It was found that the content of N and P in the FPR substrate was the highest among the three substrates, which may lead to the excessively vigorous vegetative growth of the plant, thus inhibiting fruit development to some extent. In this study, the fruit antioxidant activity was determined as DPPH and FRAP. The higher DPPH values in the FPB and FBR treatments may be related to the accumulation of antioxidant substances in the fruit, such as phenols, anthocyanins, and flavonoids [52].

Metabolomics can provide definitive information about plant phenotypes under specific environmental conditions [53]. Changes at the upstream gene and protein levels can be amplified in many downstream metabolites and make these changes more easily observed, so metabolomics can better explain the interaction between biological systems and their environment [17]. For instance, Yang et al. analyzed the effects of different formula ratios on the growth and development of blueberries by metabolomics techniques, and the results suggested that “flavonoid biosynthesis” was the most enriched pathway and preliminarily revealed the relationship between flavonoids and blueberry growth and development [17]. Yang et al. found that five different anthocyanin metabolites were the main pigment types among differently colored hawthorn berries [54].

In this study, 154, 258, and 224 DAMs were identified in three comparisons (FPB vs. FPR (CK), FPB vs. FBR, and FBR vs. FPR (CK), respectively) by metabolomic analysis. Comprehensive analysis revealed that different cultivation substrate treatments had a greater impact on the metabolism of flavonoids, carbohydrates, and amino acids than on that of other substances, indicating that these substances were particularly related to the formation of fruit quality. The content of four flavonoids (tectorigenin, naringenin chalcone, trifolirhizin, and galangin) of the FPB group was greatly increased compared with that in the FPR (CK) and FBR treatment groups. In the FBR vs. FPR (CK) comparison, most flavonoids were upregulated, with procyanidin A2, camelliaside A, and morin hydrate levels rising most significantly, which was likely the main reason for the increase in flavonoid content in the FBR treatment group. The results indicated that 50:50 pine bark/rice husk substrate was beneficial for the synthesis of flavonoids. Previous studies on the vegetative growth stage of blueberry plants found that the water retention performance of this substrate formula was not ideal, exposing the blueberries to moderate water stress, which may have further promoted the synthesis of flavonoids in the fruit [17]. As an aromatic amino acid, Phe is a precursor to the synthesis of anthocyanins and flavonoids, which play an important role in their biosynthesis process [55]. This study revealed that the content of Phe in the FBR-treated group fruit increased 2.33-fold compared with that in the FPR (CK) group, which may promote the synthesis of polyphenols in the FBR treatment group. Additionally, the highest K content in the FBR substrate may also play an important role in the metabolism of amino acids in the fruit, since K is closely related to the biosynthesis, transformation, and distribution of metabolites [56,57]. Sugar is an indispensable part of the plant growth process; it not only provides a carbon skeleton for plants, but also increases the resistance of plants [10,58]. In fruits, anthocyanin and flavonol synthesis requires the addition of sugar moieties by glycosylation [58]. The levels of sucrose, D-fructose 1,6-bisphosphate, and salidroside in the FPB treatment group were significantly upregulated compared to those in the FPR (CK) and FBR treatment groups, and they may have made important contributions to the carbon supply during fruit development. The carbon metabolism, amino acid metabolism, and secondary metabolite metabolism of blueberry fruits differed significantly among different cultivation substrate treatments. Our results provide a reference for the selection of blueberry cultivation substrates and information on the factors that cause fruit quality differences.

## 5. Conclusions

In summary, this study showed that the FPB treatment had the greatest influence on the fruit size, color, DPPH value, sugar content, acid content, solidity-acid ratio, anthocyanin content, and ellagic acid content. The FBR treatment group had the highest contents of polyphenols and flavonoids and had a higher solidity-acid ratio and anthocyanin content than the other groups. In the FPR (CK) group, the free radical level, SOD and POD activities, fruit firmness, and pectin content of the fruit were the highest, while the solidity-acid ratio and anthocyanin and flavonoid contents of the fruit were the lowest. A total of 839 metabolites were identified in this study, with the most DAMs detected in the FPB vs. FBR comparison. Flavonoids, carbohydrates, and amino acid metabolites varied most significantly. Sucrose, D-fructose 1,6-bisphosphate, salidroside, tectorigenin, naringenin chalcone, trifolirhizin, and galangin were significantly upregulated in the FPB treatment group. The contents of procyanidin A2, camelliaside A, morin hydrate, and Phe were significantly upregulated in the FBR treatment group. It was suggested that the substrate formulation of the FPB and FBR treatment groups promoted the development of blueberry fruit and the synthesis of bioactive substances in fruit. These results will facilitate the selection of soilless cultivation substrates for blueberries. FPB substrate cultivation should be chosen if the appearance, shelf-life, flavor, and anthocyanin content of the fruit are prioritized. These results improve our understanding of the main active substances that affect blueberry fruit quality and will facilitate the production of high-quality blueberries in future substrate cultivation efforts.

## Figures and Tables

**Table 1:** Eluent gradient.

Time (min)	A%	B%
0	98	2
1.5	98	2
3	15	85
10	0	100
10.1	98	2
11	98	2
12	98	2

**Table 2:** Mass spectrometry parameters.

Parameters	Positive Ion	Negative Ion
Mass Scan Range	100–1500	100–1500
Spray Voltage (kV)	3.5	3.5
Sheath Gas Flow Rate (psi)	35	35
Aux Gas Flow Rate (L/min)	10	10
Capillary Temperature (°C)	320	320
S-lens RF Level	60	60
Aux Gas Heater Temperature (°C)	350	350

**Table 3:** Effects of soilless substrates on the weight, size, and color of blueberry fruits.

Treatment	Fruit Weight (g)	Transverse Diameter of Fruit (mm)	Vertical Diameter of Fruit (mm)	L *	a *	b *
FPR (CK)	1.09 ± 0.13 b	13.23 ± 0.66 a	10.88 ± 0.61 b	34.10 ± 1.84 c	−0.17 ± 0.08 b	−13.44 ± 1.00 a
FPB	1.29 ± 0.17 a	13.40 ± 0.77 a	11.90 ± 0.59 a	38.44 ± 2.31 a	−0.18 ± 0.20 b	−15.21 ± 1.13 b
FBR	1.14 ± 0.12 b	12.91 ± 0.92 a	11.85 ± 0.71 a	36.19 ± 1.59 b	−0.01 ± 0.11 a	−14.75 ± 0.78 b

Values are shown as the mean ± SD (*n* = 3). Means followed by different letters are significantly different between the treatments according to Tukey’s tests (*p* < 0.05).

**Table 4:** Effects of soilless substrates on fruit firmness, total pectin content, the stem scar size, and the drip loss.

Treatment	Fruit Firmness (kg cm^−2^)	Total Pectin Content (g kg^−1^ FW)	Stem Scar Size (mm)	Drip Loss (%)
FPR (CK)	2.71 ± 0.29 a	2.92 ± 0.03 a	3.02 ± 0.12 a	4.71 ± 0.44 b
FPB	2.47 ± 0.21 b	2.73 ± 0.09 ab	3.11 ± 0.12 a	5.38 ± 0.35 b
FBR	2.43 ± 0.23 b	2.42 ± 0.20 b	3.06 ± 0.12 a	6.31 ± 0.08 a

Values are shown as the mean ± SD (*n* = 3). Means followed by different letters are significantly different between the treatments according to Tukey’s tests (*p* < 0.05).

## Data Availability

Data are contained within the article and Supplementary Materials.

## Supplementary Materials

The following supporting information can be downloaded at: https://www.mdpi.com/article/10.3390/foods11243965/s1, Figure S1: Functional and categorical annotation of identified metabolites using the KEGG, HMDB, and LIPID MAPS databases; Figure S2: Principal component analysis (PCA) of metabolites in blueberry fruits under three different soilless substrate treatments; Figure S3: Correlation analysis of the top 20 differentially abundant metabolites (DAMs) identified from the three comparisons (FPB vs. FPR (CK), FPB vs. FBR, and FBR vs. FPR (CK)) in positive and negative ion modes. The DAMs were identified by the criteria of *p* value (*p* < 0.05), with red representing a positive correlation and blue representing a negative correlation; Table S1: Metabolites identified in all samples.
