# Supplementary material for: Integrated Physiological and Metabolomic Analyses Reveal the Differences in the Fruit Quality of the Blueberry Cultivated in Three Soilless Substrates
Source: Foods. 2022 Dec 7;11(24):3965. doi: 10.3390/foods11243965 (PMC9777891; doi:10.3390/foods11243965)
Supplement: Supplementary file 1 [file foods-11-03965-s001.zip › foods-2030454-supplementary.pdf]

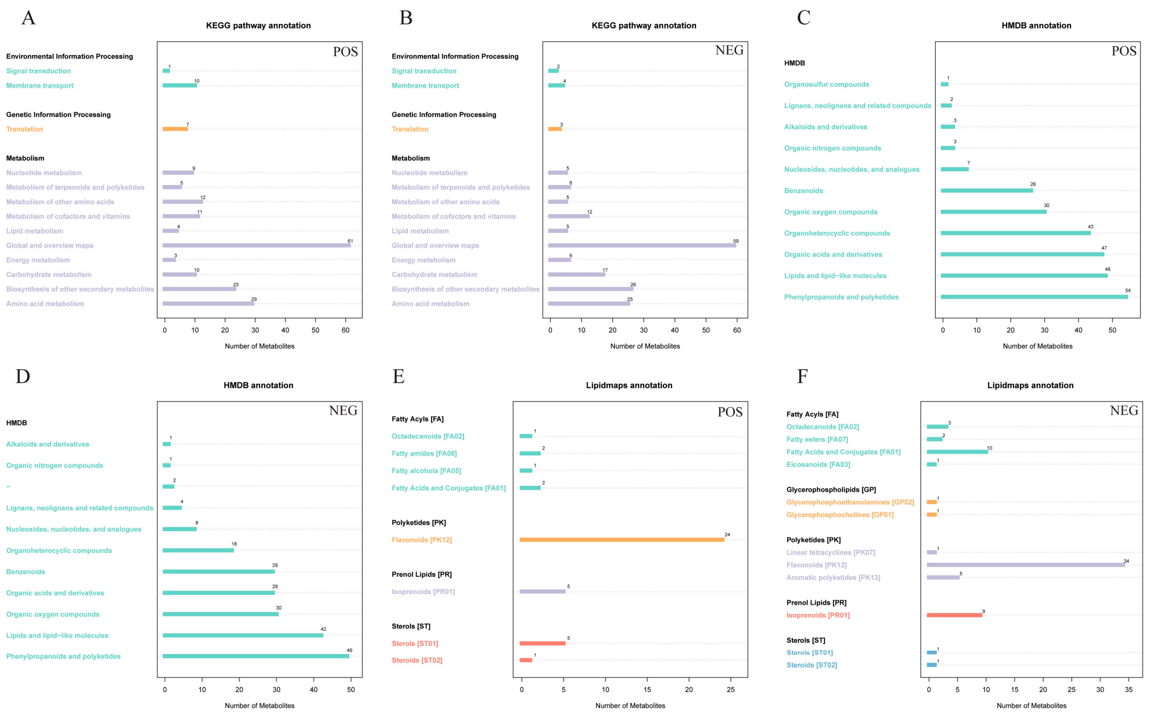

Figure S1: Functional and categorical annotation of identified metabolites using the KEGG, HMDB, and LIPID MAPS databases; KEGG annotation in positive (**A**) and negative (**B**) ionization modes; HMDB annotation in positive (**C**) and negative (**D**) ionization modes; LIPID MAPS annotation in positive (**E**) and negative (**F**) ionization modes.

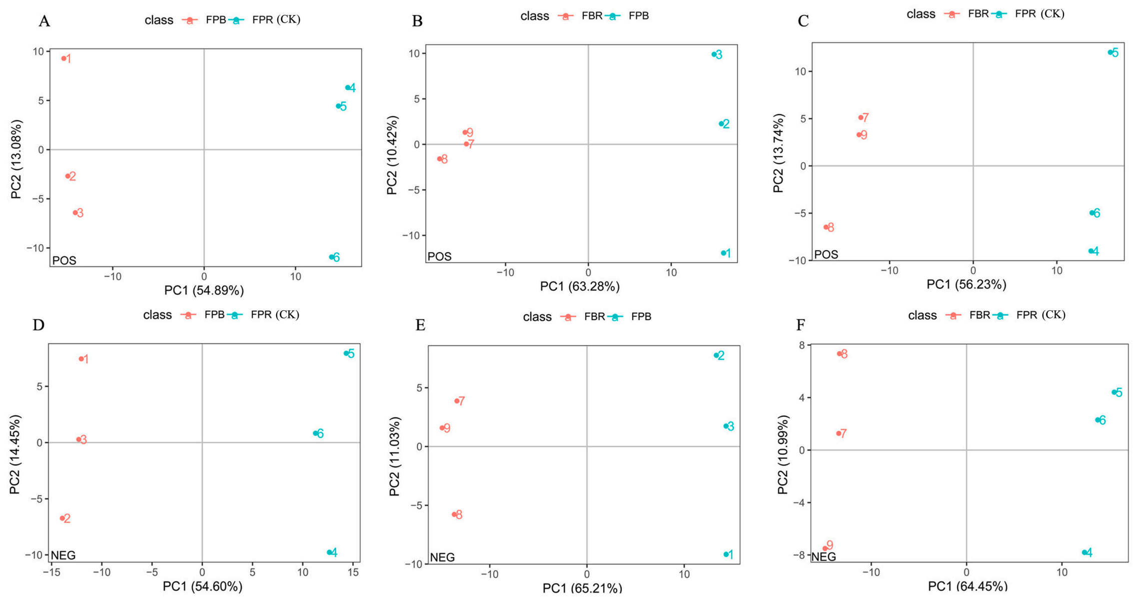

Figure S2: Principal component analysis (PCA) of metabolites in blueberry fruits under three different soilless substrate treatments; The comparisons in positive (FPB vs. FPR (CK) (**A**), FPB vs. FBR (**B**), and FBR vs. FPR (CK) (**C**)) and negative (FPB vs. FPR (CK) (**D**), FPB vs. FBR (**E**), and FBR vs. FPR (CK) (**F**)) ionization modes.

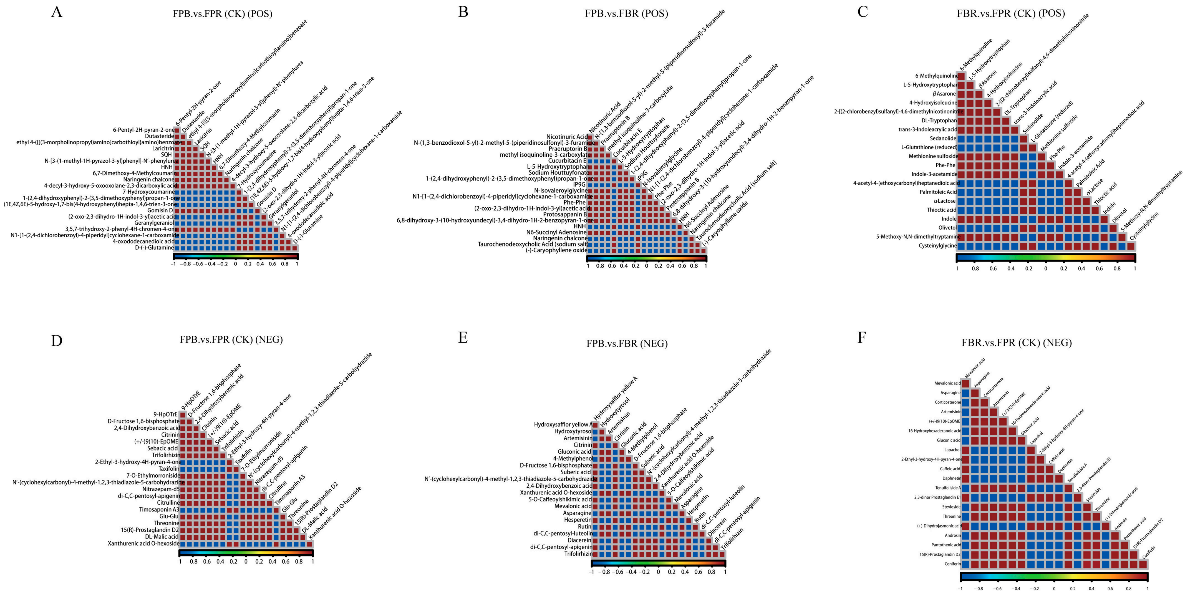

Figure S3: Correlation analysis of the top 20 differentially abundant metabolites (DAMs) identified from the three comparisons in positive (FPB vs. FPR (CK) (**A**), FPB vs. FBR (**B**), and FBR vs. FPR (CK) (**C**)) and negative (FPB vs. FPR (CK) (**D**), FPB vs. FBR (**E**), and FBR vs. FPR (CK) (**F**)) ion modes. The DAMs were identified by the criteria of *p* value ($p < 0.05$), with red representing a positive correlation and blue representing a negative correlation.
