# Supplementary figures and images for: Integrated Physiological and Metabolomic Analyses Reveal the Differences in the Fruit Quality of the Blueberry Cultivated in Three Soilless Substrates
Source: Foods. 2022 Dec 7;11(24):3965. doi: 10.3390/foods11243965 (PMC9777891; doi:10.3390/foods11243965)

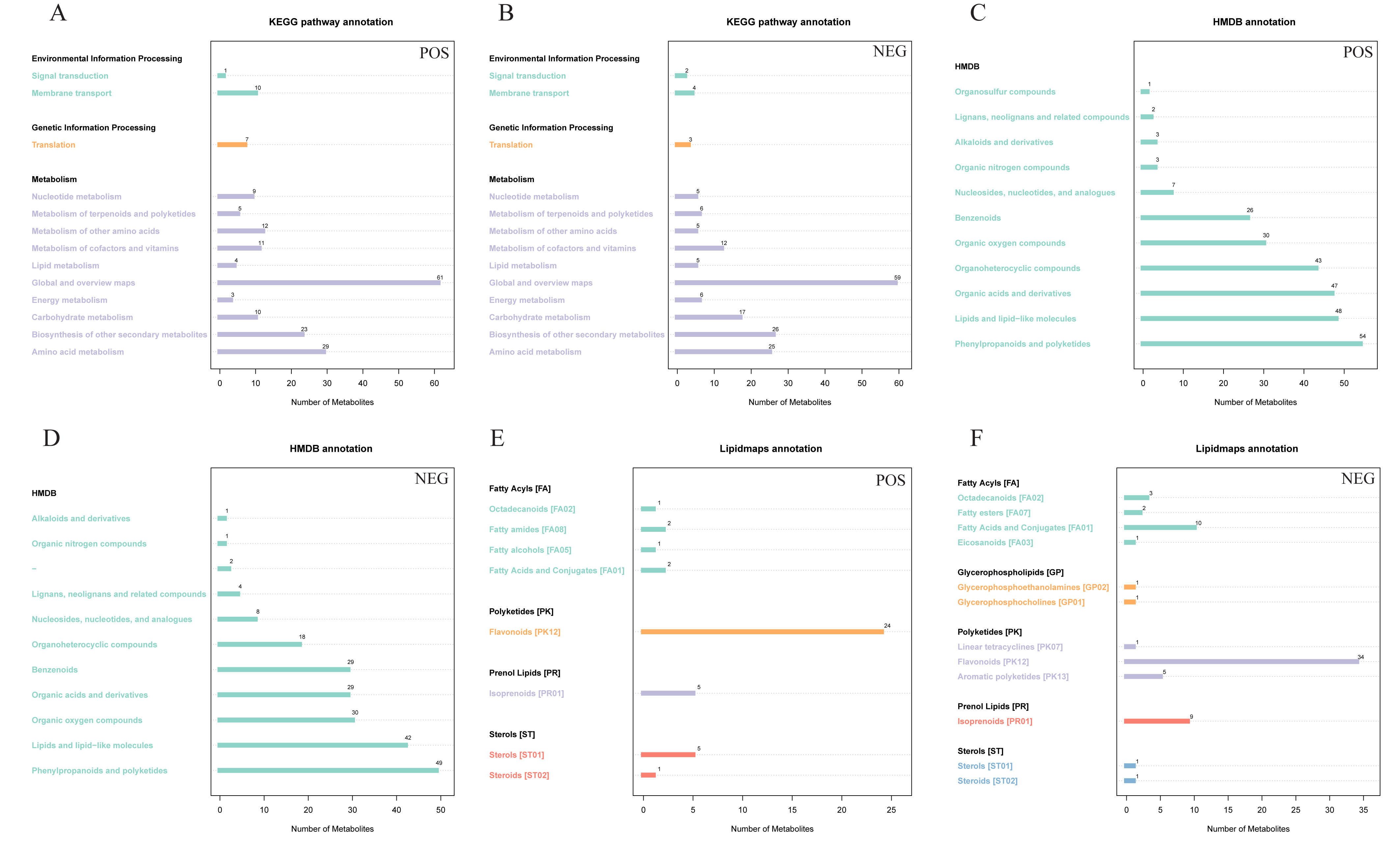

Supplement: Supplementary file 1 [file foods-11-03965-s001.zip › Supplementry Figure S1.jpg]

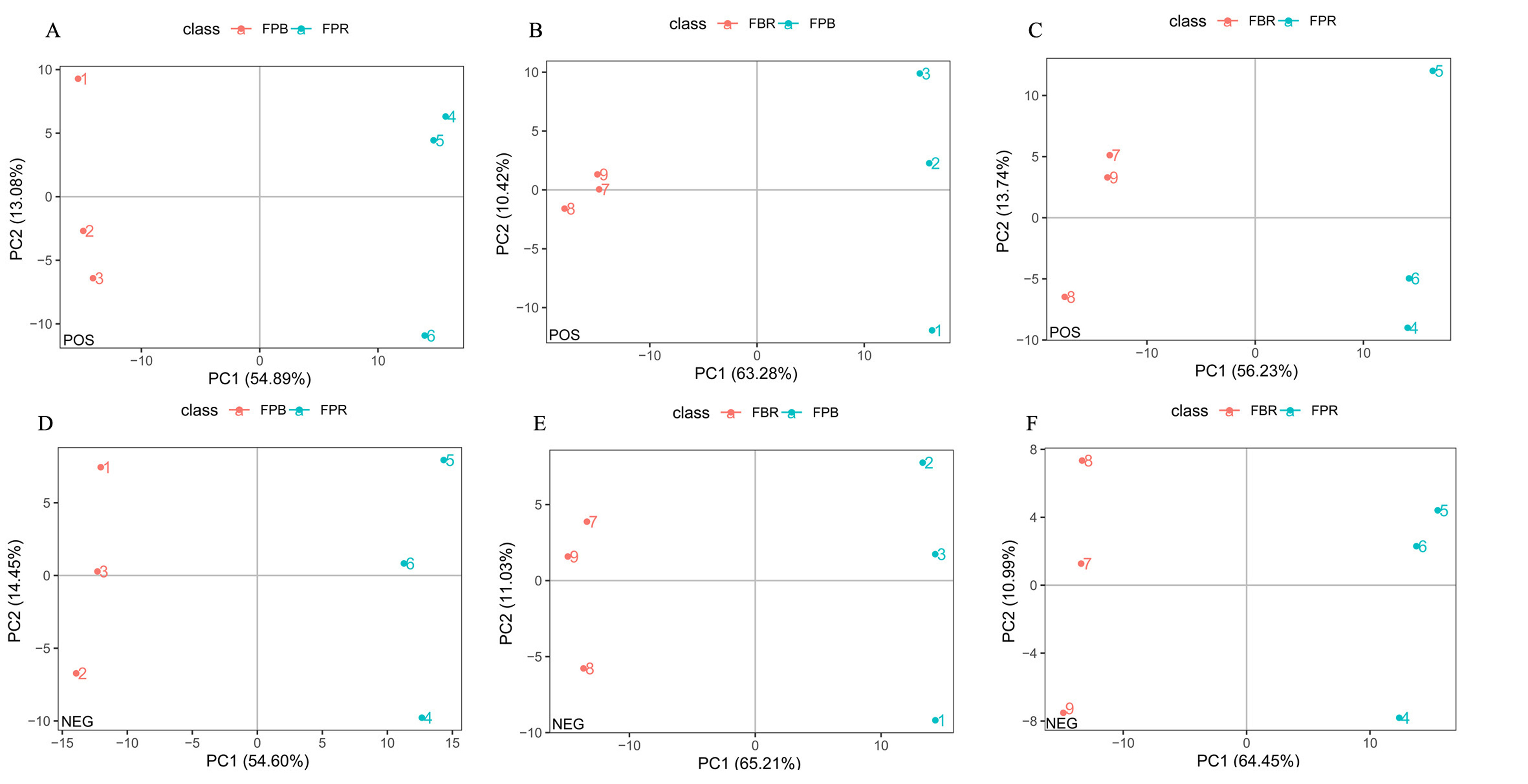

Supplement: Supplementary file 1 [file foods-11-03965-s001.zip › Supplementry Figure S2.jpg]

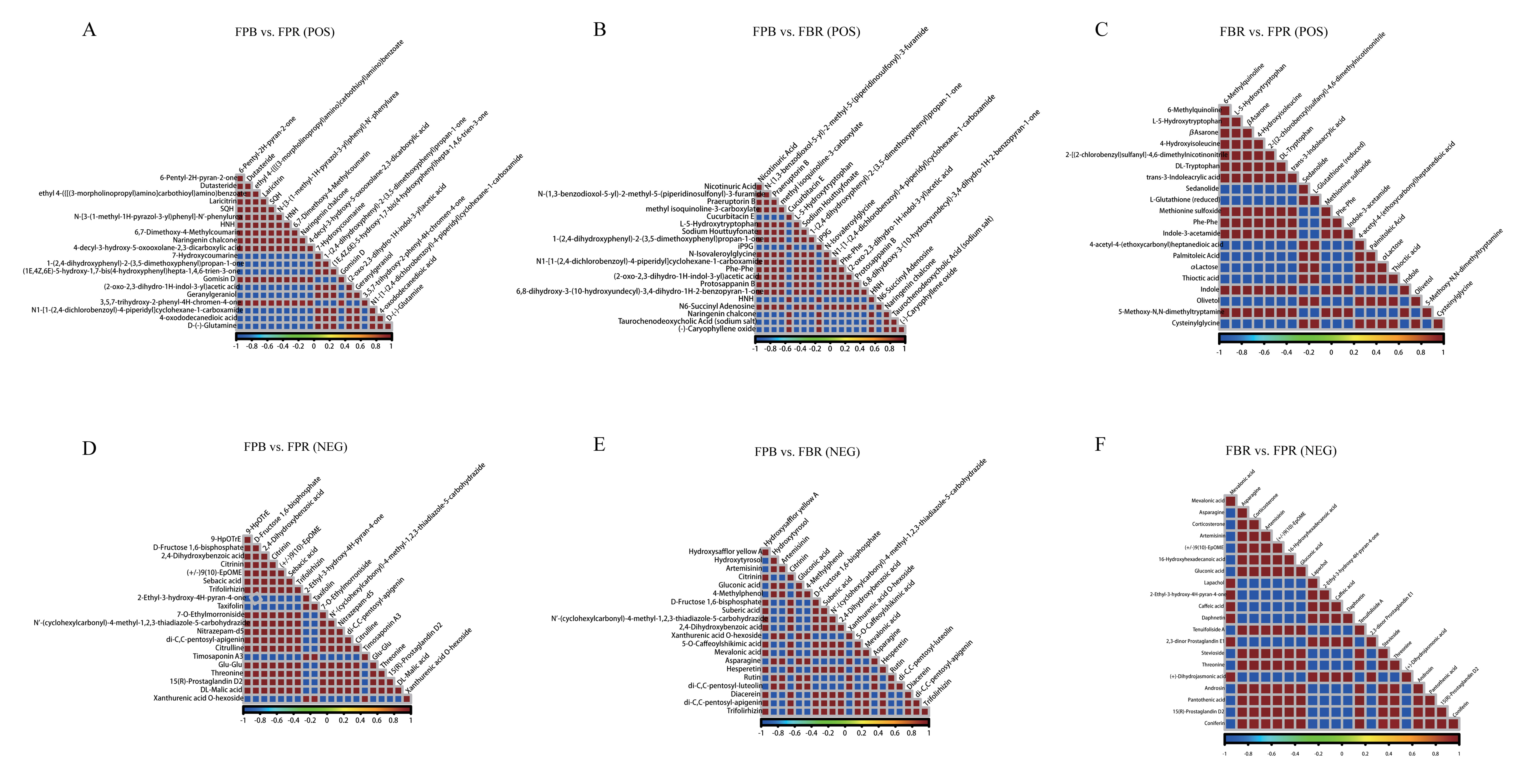

Supplement: Supplementary file 1 [file foods-11-03965-s001.zip › Supplementry Figure S3.jpg]
